# Biomimetic Radical Chemistry and Applications

**DOI:** 10.3390/molecules27072042

**Published:** 2022-03-22

**Authors:** Chryssostomos Chatgilialoglu

**Affiliations:** 1 ISOF, Consiglio Nazionale delle Ricerche, 40129 Bologna, Italy; chrys@isof.cnr.it; Tel.: +39-051-639-8309; 2 Center of Advanced Technologies, Adam Mickiewicz University, 61-712 Poznań, Poland

## Abstract

Some of the most interesting aspects of free radical chemistry that emerged in the last two decades are radical enzyme mechanisms, cell signaling cascades, antioxidant activities, and free radical-induced damage of biomolecules. In addition, identification of modified biomolecules opened the way for the evaluation of in vivo damage through biomarkers. When studying free radical-based chemical mechanisms, it is very important to establish biomimetic models, which allow the experiments to be performed in a simplified environment, but suitably designed to be in strict connection with cellular conditions. The 28 papers (11 reviews and 17 articles) published in the two Special Issues of *Molecules* on “Biomimetic Radical Chemistry and Applications (2019 and 2021)” show a remarkable range of research in this area. The biomimetic approach is presented with new insights and reviews of the current knowledge in the field of radical-based processes relevant to health, such as biomolecular damages and repair, signaling and biomarkers, biotechnological applications, and novel synthetic approaches.

## 1. Introduction

Free radicals have attracted considerable attention in various research areas, including organic synthesis, material science, atmospheric chemistry, radiation chemistry, pharmacology, biology, and medicine [1]. Free radicals are generated in the biological environment as a result of normal intracellular metabolism and function as physiological signaling species that participate in the modulation of apoptosis, stress responses, and proliferation [2]. The enormous importance of free radical chemistry for a variety of biological events, including ageing and inflammation, has motivated studies to understanding the related mechanistic steps at the molecular level. Therefore, the estimation of the type and extent of damages, as well as mechanisms and efficiency of protective and repair systems, are important subjects in life sciences.

**Figure 1:**
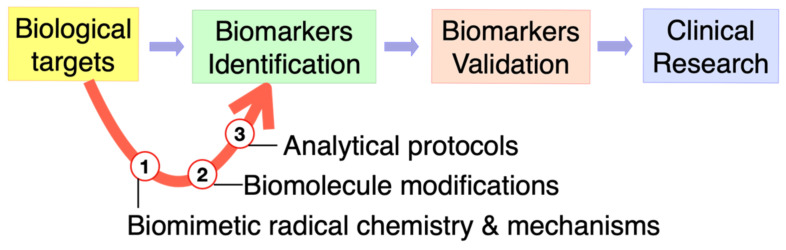
Omics technologies and the role of biomimetic radical chemistry in biomarkers discovery.

Modelling free radical reactivity of biological systems is a crucial research area. Some of the most interesting aspects of free radical chemistry that have emerged in the last two decades are radical enzyme mechanisms, cell signaling cascades, antioxidant activities, and biomarkers of free radical damage to biomolecules [1,2]. In the latter case, identification of modified biomolecules has a diagnostic value for the evaluation of in vivo damages. When studying free radical-based chemical mechanisms, biomimetic chemistry and the design of related biomimetic models come into play to perform experiments in a controlled environment, strictly connected with cellular conditions. Figure 1 shows the connections of biomarkers identification through biomimetic radical chemistry and analytical protocols of biomolecule modifications, as well as their extension to clinical research such as ageing, inflammation, cancer, obesity, and other pathologies.

The papers published in the two Special Issues of *Molecules* on “Biomimetic Radical Chemistry and Applications (2019 and 2021)” show the strong interdisciplinary context with a remarkable range of research in this area. Several subjects are presented, with 17 articles and 11 reviews written by specialists in the fields. 

## 2. Reactive Oxygen/Nitrogen Species (ROS/RNS) Network

The free radical history in biology and medicine up to 1990 had three main entries: the ‘free radical theory of aging’ based on the damage and recycling involving reactive oxygen species (ROS) [3], the properties of the enzyme superoxide dismutase (SOD) [4], and the antioxidant network including the role of vitamins [2]. Now days, it is well documented that ROS, reactive nitrogen species (RNS), and reactive sulfur species (RSS) are produced in a wide range of physiological processes and are also responsible for a variety of pathological processes. Indeed, the overproduction of ROS/RNS/RSS has been linked with the etiology of various diseases, and antioxidant defense mechanisms are essential to protect against them [5,6]. 

**Figure 2:**
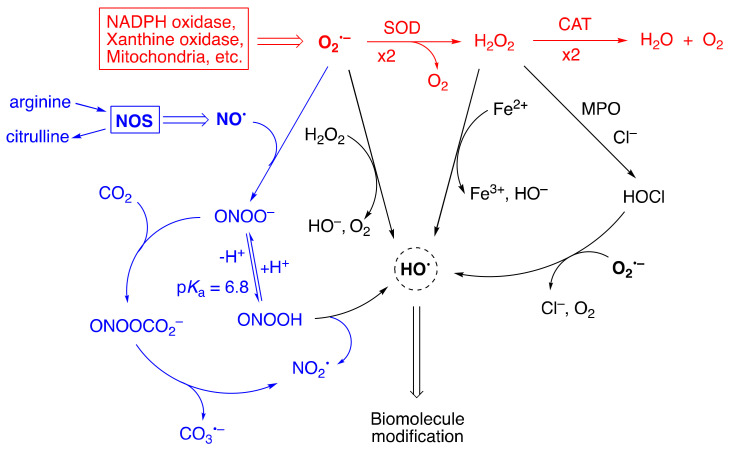
Pathways of interaction of superoxide and nitric oxide in biological systems. NOS: nitric oxide synthase; SOD: superoxide dismutase; CAT: catalase; MPO: myeloperoxidase.

Figure 2 summarizes the main feature of the ROS/RNS network, including molecules such as hydrogen peroxide (H_2_O_2_), hypochlorous acid (HOCl) and peroxynitrite (ONOO^−^), as well as radicals such as superoxide radical anion (O_2_^•−^), nitric oxide (NO^•^), hydroxyl radical (HO^•^), nitrogen dioxide (NO_2_^•^), and the carbonate radical anion (CO_3_^•−^). Aerobic life would not be possible without the enzymes SODs and catalase (CAT) that transform superoxide to water and oxygen. Nitric oxide synthase (NOS) is a class of enzymes that induce the formation of nitric oxide (NO^•^). Under physiological conditions, concentrations of ∼0.1 nM O_2_^•−^ and ∼10 nM NO^•^ play a role in regulating the activation of transcription factors, cell proliferation and apoptosis. During the inflammatory response, their concentration can increase up to a 100-fold excess. These two radicals are the precursors of a variety of other reactive species. 

Figure 2 shows the main pathways by which other biologically important free radicals can be produced, either via H_2_O_2_ or as a consequence of ONOO^−^ formation from O_2_^•−^ and NO^•^ [1,2,5,6]. H_2_O_2_ is at the crossroad of several pathways; the main ones are reported in Figure 2. Myeloperoxidase (MPO) uses H_2_O_2_ and anions like Cl^−^, Br^−^, SCN^−^ and NO_2_^−^ to generate hypochlorous acid (HOCl) or HOBr, HOSCN and NO_2_^•^, respectively. H_2_O_2_ transformation to highly reactive HO^•^ occurs by the Fenton reaction (Fe^2+^ and H_2_O_2_), the Haber–Weiss reaction (O_2_^•−^ and H_2_O_2_), and reduction of previous formed HOCl by O_2_^•−^. ONOO^−^ exists in equilibrium with its protonated form (p*K*_a_ = 6.6), which spontaneously decomposes to NO_2_^•^ and HO^•^. Other ONOO^−^ also reacts with CO_2_ and the resulting adduct rapidly decomposes to NO_2_^•^ and CO_3_^•−^. Although O_2_^•−^ is very unreactive in typical free radical reactions, such as hydrogen atom abstraction or addition, its successors generate the most reactive HO^•^. The diffusion distance of HO^•^ is very small because of their high reactivity with all types of biomolecules and, consequently, there is a low probability to be intercepted by antioxidants [7].

## 3. Brief Overview of the Two Special Issues

### 3.1. Targeting DNA Damage and Repair 

Three reviews dealing with oxidative DNA damage and repair appeared in the two Special Issues [8,9,10]. One electron oxidation gives rise to a radical cation whose charge (hole) can migrate through DNA covering several hundreds of Å, eventually leading to irreversible oxidative damage; an overview of work on the dynamics of hole transfer in DNA is reported [8]. Among the four common DNA bases (A, G, T, and C), G is the most readily oxidized to the G radical cation (G^•+^), which is also the putative initial intermediate in the oxidative DNA damage. Upon formation of G^•+^, fast deprotonation occurs by loss of a proton to give the guanyl radical G(-H)^•^, also named the guanine radical or neutral guanine radical. An overview of the one-electron oxidation of the GC pair and the complex mechanism of deprotonation vs. hydration steps of GC^•+^ pair is given. The role of the two G(-H)^•^ tautomers in single- and double-stranded oligonucleotides and the G-quadruplex, the supramolecular arrangement that attracts interest for its biological consequences, are discussed, including the importance of biomarkers of guanine DNA damage [9]. To maintain genomic stability and integrity, double-strand DNA has to be replicated in a strictly regulated manner, ensuring the accuracy of its copy number and its integrity. DNA damage-induced replication stress, the formation of DNA secondary structures, peculiar epigenetic modifications and cellular responses to the stress and their impact on the instability of the genome and epigenome, mainly in eukaryotic cells, have been summarized [10]. 

Six articles report new experimental data on targeting DNA damage [11,12,13,14,15,16]. Guanine radicals generated in single, double, and G-quadruplex oligonucleotides are studied by nanosecond transient absorption spectroscopy [11]. The time needed to establish electronic resonant conditions for charge transfer in oxidized DNA has been evaluated by molecular dynamics simulations followed by QM/MM computations, which include counterions and a realistic solvation shell [12]. Among the reactive oxygen species (ROS), the hydroxyl radical (HO^•^) is the most reactive toward any biomolecule including DNA (cf. Figure 2). Although the majority of the purine DNA lesions like 8-oxo-purine (8-oxo-Pu) are generated by various ROS (including HO^•^), the formation of 5′,8-cyclopurine (cPu) lesions in vitro and in vivo relies exclusively on the HO^•^ attack. Indeed, recent research focused on the purine DNA damage by HO^•^ with emphasis on mechanistic aspects for the various lesion formation and their interconnections [17]. New insights into the reaction paths of HO^•^ with genetic material and the formation of 8-oxo-Pu and cPu lesions vs. oxygen concentration have been reported in detail [13,18]. It is worth mentioning a recent article on inflammatory bowel disorders (IBD), showing the role of cPu as a biomarker [19]. Radiosensitizing properties of substituted uridines are of great importance for radiotherapy. The influence of the type of halogen atom in the radio-sensitizing properties of 5-halo-4-thio-2-deoxyuridine has been addressed; contrary to the 5-iodo derivative that is an efficient agent, the 5-bromo does not show radio-sensitizing properties at all [14]. Several classes of copper complexes prepared in the past were explored for their chemical nuclease activity both biologically and chemically [20]. Two studies include the mechanism of copper artificial metallo-nuclease to induce superoxide-mediated cleavage via the minor groove [15], and the DNA binding and quantitative cleavage activity of the [Cu(TPMA)(*N*,*N*)]^2+^ class (where TPMA = tris-2-pyridylmethylamine) using a DNA electrochemical biosensor [16]. 

### 3.2. Protein Modifications and Enzymatic Activity 

In the area of proteins, four reviews contribute to the two Special Issues [21,22,23,24]. The formation of covalently linked peptides and proteins plays a key role in many biological processes, both physiologically and pathologically. In one review, it is summarized: the spectrum of crosslinks currently known to be formed on proteins, including the mechanisms of their formation, experimental approaches to the detection, and identification and characterization of these species [21]. Reversible crosslinks, driven by the formation of disulfide bridges, appear to play a key role in cell signaling events, primarily as a result of reversible thiol–disulfide switches or related species. Irreversible protein crosslinks are mostly unwanted processes, occurring during metabolism, that can accumulate in aging or have been associated with the onset and development of pathological conditions and human diseases. Their recognition as reliable biomarkers of several pathologies, particularly neurodegenerative disorders, is an important field of molecular diagnostics in medicine [21,25]. Another review provides a thorough description of the role of phosphatidylethanolamine-derived protein adducts and effects on membrane properties [22]. The aim was to highlight this new area of research and to encourage a more nuanced investigation of the complex nature of the new lipid-mediated mechanism in the modification of membrane protein functions under oxidative stress.

When proteins are pharmaceutical compounds, such as insulin or human growth hormone, their degradation can occur by radical species that are generated from pharmaceutical excipients. Polysorbate is prone to generate peroxyl radicals that can trigger oxidative degradation [26]. In this review, the involvement of thiyl radicals in pharmaceutical protein degradation through hydrogen atom transfer, electron transfer, and addition reactions is reported [23]. Another review reports the work done in the chemical labelling of proteins using biomimetic radical chemistry [24]. This is inspired by the occurrence of radical reactions in an aqueous environment, such as for enzymatic catalysis or photoreactions. Such reactivity occurs selectively on specific amino acids (nucleophilic residues such as lysine or cysteine) by means of electrophilic compounds that allow site-selective protein labeling. This is an important field of chemical biology.

An overview of reductive dihydroxylation catalyzed by IspH, an enzyme involved in the biosynthesis of isoprenoids, has been reviewed [27]. IspH is an oxygen sensitive [4Fe-4S] metalloenzyme that catalyzes 2H^+^/2e^−^ reductions and water elimination by involving non-conventional bioinorganic and bioorganometallic intermediates. This review focuses on the IspH mechanism, discussing the results that have been obtained in the last decades using an approach combining chemistry, enzymology, crystallography, spectroscopies, and docking calculations. A section about the inhibitors of IspH discovered up to now is also reported. The presented results constitute a useful and rational approach to inaugurate the design and development of new potential chemotherapeutics against pathogenic organisms.

Three original articles reported research related to biotechnological applications of proteins: (i) Therapeutic uses of natural peptide somatostatin is limited by its very short biological half-life of 1–2 min. The entrapment of this peptide in a lipid formulation allowed to retard release in aqueous medium and human plasma. Furthermore, a new radical reactivity was discovered arising from the interaction between this sulfur-containing peptide and its liposomal formulation [28]. (ii) Physico-chemical evidence showed that riboflavin together with hyaluronic acid play a role in the treatment of corneal cross-linking treatment of keratoconus by UVA light. Spin trapping experiments on collagen/hyaluronic acid/riboflavin solutions evidenced the formation of reactive oxygen species (ROS) by electron paramagnetic resonance measurements. Riboflavin under UVA irradiation generates ROS that can induce damages, whereas hyaluronic acid has a protecting role [29]. (iii) The formation and stabilization of gold nanoparticles in bovine serum albumin (BSA) solution has been discovered. Physico-chemical studies showed that the size of nanoparticles increases slowly with time, resulting in nanoparticles of different morphologies, and the stabilization is obtained through the interaction of sulfur-containing amino acid residues of albumin [30].

### 3.3. Various Bioinspired Mechanistic Studies and Applications 

Three reviews deal with confocal microscopy-based detection [31], mechanistic studies of methionine (Met) oxidation [32], and recent applications of chemiluminescence (CL) [33]. One of them focuses on confocal microscopy-based detection of profound alterations in the plasma membrane, membranes of insulin granules and lipid droplets in single beta cells under various nutritional load conditions. The combination of whole cell lipidomics analysis and single cell confocal imaging of fluidity and micropolarity provides insight into stress-induced lipid turnover in subcellular organelles of pancreatic beta cells [31]. 

Oxidation of methionine (Met) is an important reaction that plays a key role in protein modifications during oxidative stress and aging. An overview of the transient species detection in one-electron oxidation of Met derivatives by various time-resolved techniques is presented [32]. Mechanistic aspects of Met oxidation in various structural environments (e.g., peptide) and at various pH by one-electron oxidants (including HO^•^ radical) are summarized and discussed. Neighboring group participation seems to be an essential parameter which controls one-electron oxidation of methionine. The observed transient species are precursors of final products [32].

The phenomenon of chemiluminescence (CL) can take place both in natural and artificial chemical systems and has been utilized in a variety of applications. A review reports on recent research in this area [33]. In this context, the CL role in the development of efficient therapeutic platforms is also discussed in relation to the reactive oxygen species (ROS) and singlet oxygen (^1^O_2_) produced as final products. The CL prospects in imaging, biomimetic organic and radical chemistry, and therapeutics are critically presented in respect to the persisting challenges and limitations of the existing strategies to date [33].

Heme iron and non-heme dimanganese catalases (CAT) protect biological systems against oxidative damage caused by hydrogen peroxide (cf. Figure 2). In order to gain more insight into the mechanism of these curious enzyme reactions, two original articles reported on the metal complexes as catalase mimics: a mononuclear non-heme oxoiron(IV) complex mediated H_2_O_2_ dismutation into O_2_ and H_2_O in aqueous solution [34], and a non-heme diiron-peroxo complex which shows a catalase-like reactivity [35].

The conversion of ribonucleosides to 2′-deoxyribonucleosides is catalyzed by ribonucleoside reductase enzymes in nature. One of the key steps in this complex radical mechanism is the reduction of the 3′-ketodeoxynucleotide by a pair of cysteine residues, providing the electrons via a disulfide radical anion (RSSR^•−^) in the active site of the enzyme [36]. Experimental conditions were found to obtain the bioinspired conversion of ketones to corresponding alcohols with high-yield by the intermediacy of disulfide radical anion of cysteine (CysSSCys)^•−^ in water [37].

Mechanistic studies of radical processes in pharmacological applications, which also inspire biological mechanisms, are represented by various original articles: the oxidation of 8-thioguanosine and 2-thiouracil by photolytic and radiolytic conditions [38,39]; bioinspired radical-based synthetic strategies toward anomeric spironucleosides as potential inhibitors of glycogen phosphorylase and for the preparation of azido-derivatives via a radical azidoalkylation of alkenes [40,41]; and the synthesis of two new iron-porphyrin-based catalysts inspired by naturally occurring proteins, such as horseradish peroxidase, hemoglobin, and cytochrome P450, tested for atom transfer radical polymerization (ATRP), obtaining polymers with specific properties [42]. 

## 4. Conclusions

The two Special Issues give the reader a wide overview of biomimetic radical chemistry, where molecular mechanisms have been defined and molecular libraries of products are also developed to be used for the discovery of some relevant biological processes. The biomimetic approach is a convenient tool, since achievements in free radical mechanisms can be easily transferred to a better comprehension of the radical-based biological pathways in living organisms, triggering advancements in health and diseases. In addition, identification of modified biomolecules paves the way for molecular libraries and the evaluation of in vivo damage through biomarkers. 

The two Special Issues cover aspects of free radical chemistry in biological events, revealed using biomimetic chemical models. These include: catalytic pathways and mechanisms of radical enzymes, prebiotic chemistry, radical-induced DNA lesions or protein modifications, with further development concerning analytical protocols, repair processes, biological consequences, lipid peroxidation and isomerization, and defense systems based on antioxidants, as well as bio-inspired synthetic strategies.

## Data Availability

Not applicable.
